# Females Paired with New and Heavy Mates Reduce Intra-Clutch Differences in Resource Allocation

**DOI:** 10.1371/journal.pone.0072136

**Published:** 2013-08-15

**Authors:** Maud Poisbleau, Nina Dehnhard, Laurent Demongin, Charline Parenteau, Petra Quillfeldt, Marcel Eens

**Affiliations:** 1 Department of Biology - Ethology, University of Antwerp, Wilrijk, Belgium; 2 Max Planck Institute for Ornithology, Vogelwarte Radolfzell, Radolfzell, Germany; 3 Centre d’Etudes Biologiques de Chizé, Centre National de la Recherche Scientifique, Villiers en Bois, France; 4 Behavioural Ecology & Ecophysiology group, Justus Liebig University, Giessen, Germany; Arizona State University, United States of America

## Abstract

Reproductive investment affects both offspring and parental fitness and influences the evolution of life histories. Females may vary their overall primary reproductive effort in relation to the phenotypic characteristics of their mate. However, the effects of male quality on differential resource allocation within clutches have been largely neglected despite the potential implications for mate choice and population dynamics, especially in species exhibiting biparental care and brood reduction. Female southern rockhopper penguins *Eudyptes chrysocome* paired with heavy mates reduced intra-clutch variation in egg and albumen masses. Females paired with new mates also reduced intra-clutch variation in yolk androgen levels. Since both an increased mass and increased androgen concentrations positively influence chick survival under sibling competition, the chances of fledging the whole clutch are likely to be higher for newly formed pairs with heavy males than for previously formed pairs with light males. Interestingly, total clutch provisioning did not vary with male quality. We show for the first time that females vary intra-clutch variation in resource allocation according to male quality. In species with brood reduction, it may be more adaptive for females to modulate the distribution of resources within the clutch according to breeding conditions, than to change their total clutch provisioning.

## Introduction

Life-history theory predicts that individuals should adjust their reproductive investment to the fitness gain they can expect in return [1]–[3]. For instance, the amount of resources that parents invest in offspring influences offspring survival, growth and reproduction, and subsequently affects the fitness of both the offspring and the parents [1]–[3]. The differential allocation hypothesis proposes that the selection might favor individuals that allocate resources differently according to the phenotypic characteristics of their current mate and the likelihood of finding a better mate in the future [4], [5]. It has been established that females vary their primary reproductive effort [6], in egg size and composition for example, in relation to their mate’s phenotypic characteristics (reviewed in [5], [7]). Females generally lay larger and heavier eggs and clutches when paired with an attractive male than when paired with an unattractive male [8]–[10]. However, egg composition in nutrients, hormones or antibodies, for example, does not always vary consistently with male attractiveness [11], [12]. This dissimilarity could be explained by the different fitness gains expected when increasing egg size or when modulating egg composition. An increase in egg size generally enhances offspring size, growth, survival and fitness [13]–[16]. In comparison, the benefit of modifying egg composition, such as increasing yolk hormones, is more context-dependent [17]–[19]. Nevertheless, both mechanisms can have implications for mate choice and population dynamics [19]–[21].

Females may additionally adjust their resource allocation differently within a clutch according to the fitness gain they can expect in return from each egg in respect to its position in the laying sequence [13], [22]. Several studies have suggested that intra-clutch variation in resource allocation can act as an adaptive maternal effect in altricial species where modulation of competition between siblings would benefit mothers [23]–[25]. In altricial and semi-altricial species, differential intra-clutch allocation of resources can act in combination with asynchronous hatching to lead to a size hierarchy among siblings, and to a competitive disadvantage for the youngest and/or less provisioned siblings compared to older and/or more provisioned ones [26], [27]. This hierarchy is thought to optimize the fitness of the parents by facilitating brood reduction when resource availability does not enable them to raise the entire clutch successfully [28], [29]. It can therefore be argued that, in species exhibiting necessary biparental care and brood reduction, females may modulate their intra-clutch allocation of resources according to the perception they have of their mate’s abilities to perform as a father (hereafter referred as mate quality). We hypothesize that females paired with a high quality mate should provision their clutches in order to maximize the chances of raising the entire clutch. We also propose that females paired with a low quality mate should provision their clutches in order to enable a quick brood reduction if necessary, especially in species with a fixed clutch size because these females cannot modulate the number of eggs they lay.

We aimed to test these hypotheses in southern rockhopper penguins *Eudyptes chrysocome*. Like other crested penguins, they have a fixed clutch size of two eggs. The second-laid egg (B-egg) is 28% bigger and heavier than the first-laid egg (A-egg) [30] and, while incubation starts only at clutch completion, the A-egg usually hatches one day after the B-egg [31]–[33]. Although both eggs commonly hatch, the chick hatching from the A-egg (A-chick) generally dies of starvation within a few days after hatching [31], [34]. In the Falkland Islands (Malvinas), however, parents can sometimes fledge both chicks of the clutch (2.6% of the 114 nests monitored in 2006–2007 [30], [31]), and this phenomenon is, amongst others, linked to variation in egg size [30].

Southern rockhopper penguin chicks are semi-altricial and depend on biparental care for food and defense, although they have well developed down and can thermoregulate soon after hatching [35]. As for other penguin species, both parents make a huge investment in parental care. They share the 32–34 day long incubation, with the male and female present for the first shift of about 12 days, the female undertaking the second shift of approximately 11 days and the male returning to the nest for the final shift of about 14 days. Males then guard chicks for 24–26 days with the females returning with food nearly every day, after which the chicks wait in a crèche to be provisioned by both males and females [35]. Adult body mass is likely to play a crucial role in the ability to tolerate the long fasting periods that breeders have to endure during the consecutive stages of the reproductive period [36], [37]. Furthermore, individual body mass appears to be an important factor explaining desertion in penguin species [38]–[40]. Females may therefore use male body mass as a visual indicator of mate quality to detect whether males are likely to desert the nest prematurely. In penguins, both sexes are highly philopatric and also exhibit a strong social monogamy [41]. However, divorces that sometimes occur because of failures to return after winter or to breed in the previous season tend to decrease breeding success in penguins [42] as in other bird species [43], [44]. Females paired with the same mate as during the previous breeding season may therefore be more secure about crucial parameters for breeding success, such as intra-pair breeding synchrony and the previous breeding experience of their mate, than females paired with a new mate.

We therefore expected female southern rockhopper penguins to adapt their resource allocation to these two distinct indicators of mate quality: (i) whether the females were paired with the same mate as during the previous breeding season or with a new mate and (ii) male body mass. We tested their resource allocation in egg, yolk and albumen masses, as well as in yolk androgens. In penguins including southern rockhopper penguins, egg mass increases hatching size and mass, increased masses positively influence chick survival and intra-clutch dimorphism in egg mass benefits the heaviest sibling [13], [31], [45]–[47]. Moreover, elevated yolk androgen levels benefit chick growth and positively influence chick survival under sibling competition, with the chicks hatching from eggs with high yolk androgens levels growing faster and having higher survivorship than their siblings hatching from eggs with low yolk androgen levels [17]. We therefore predicted higher clutch mass and androgen provisioning when females were paired with high quality mates (i.e. same males as during the previous breeding season or high body mass males) than when females were paired with low quality mates (i.e. new or low body mass males). We also predicted a more equitable intra-clutch distribution of egg mass and yolk androgens, and consequently an increased probability of fledging two chicks, when females were paired with high quality mates. Laying dates and female body mass, as potential determinants of female investment into eggs [48], [49], were also considered in the analyses.

## Materials and Methods

### Ethical Statement

The study was performed under proper legislation of the Belgian and Flemish law and was approved by the ethical committee on animal experimentation (Ethische Commissie Dierproeven, ID numbers: 2011/44 and 2011/45). All work was conducted under a research license granted by the Environmental Planning Department of the Falkland Islands Government (Research License No: R06/2009). This license covered animal welfare in addition to collection of the egg samples. The methods that we used (nest check, adult capture, egg collection) did not cause any desertion from nestling activity or mortality. Collected eggs were replaced with eggs found outside their own nest that we considered as lost by their original parents in order to avoid affecting the breeding success of the colony as well as the birds’ physiology and behavior. We previously observed that replacement eggs hatch and give fledglings in the same proportion as eggs in non-manipulated nests (unpublished results).

### Study Site and Birds

The study was carried out at the “Settlement colony” (51°43′S, 61°17′W) on New Island, Falkland Islands (Malvinas) in September, October and November 2010. In 2010, this colony held about 7500 breeding pairs of southern rockhopper penguins. Birds mainly breed in open rocky areas fringed by tussac grass *Poa flabellata*. The breeding biology at this large colony has been described previously in Poisbleau et al. [31]. Briefly, males arrive at the colony first (early October) and establish nest sites. Females arrive a few days later, for pairing and copulation in late October/early November. Laying and hatching intervals are relatively fixed; the second egg (B-egg) is generally laid four days after the first one (A-egg), incubation starts at clutch completion but the A-egg usually hatches one day after the B-egg (reversed hatching asynchrony).

### Adult Manipulation

Since 2006, we have marked and followed approximately 600 adults in the colony. They are equipped with 23-mm glass-encapsulated electronic transponders (TIRIS, Texas Instruments, USA) implanted under the skin of the back between the scapulae. A regular check of the colony to record which animals equipped with a transponder are present at the colony provides data on the identities of the paired birds, and potentially on instances of mate fidelity or divorce between consecutive breeding seasons. A gateway system was additionally set up in September 2010, before the arrival of the first adults to the breeding colony. It recorded the transponder number of each passing penguin equipped with a transponder as well as the date and the time it crossed. Positioned on a rock ledge that formed the single pathway of the penguins breeding in the study colony, this gateway therefore recorded the individual arrival dates at the colony after the winter period. After the arrival of the first adults, we visited the colony daily, to follow the egg laying of the females equipped with a transponder.

We captured 60 females on the day they laid their A-egg (i.e. date of laying onset) and the corresponding 60 males from six to eight days after their female partner. The sex of birds was determined from bill measurements within pairs, the bill being larger in males than females [50]. It was also verified from previous determinations (morphological and genetic) according to the transponder number. After covering their head to minimize stress, we weighed each bird to the nearest 20 g with an electronic balance following Poisbleau et al. [50]. Since indices may not provide more precise indicators of body condition than body mass alone [51], we did not control for structural size in the analyses and used body masses as continuous covariates in the analyses.

### Egg Collection and Preparation

When a new A-egg was detected in one of the 60 study nests, we collected it and replaced it with a foster egg in order to avoid affecting the birds’ physiology and behavior, and thus B-egg composition. Afterwards, we checked the nest daily until the laying of the B-egg. We collected the B-egg as soon as it was detected in the study nest and replaced it with a lost egg. As incubation in rockhopper penguins typically does not start before clutch completion [35], the A-eggs were not incubated at all and the B-eggs were not incubated for longer than 24 h at collection. We therefore assumed that the levels of embryo development (if any) were very preliminary, were equal between eggs, and were unlikely to have proceeded to the point that embryonic hormones were being produced [52]. No embryo development was observed during the preparation of any of the collected eggs. In total, we collected 60 whole clutches. After collection, we weighed the eggs to the nearest 0.1 g using a digital balance and froze them whole at −20°C.

The same method was used to prepare all frozen eggs for subsequent hormone analysis [49], [53]–[55]. We first removed the shell while the egg was still frozen. Then, we separated the yolk from the albumen by taking advantage of the fact that albumen thaws more quickly than yolk. We recorded the mass of the yolk to the nearest 0.1 g using a digital balance. Since hormone concentrations are not homogeneous within the yolk [56], [57], we carefully homogenized the yolk by swirling it with a mini-spatula. We therefore obtained a yolk sample representative of the whole yolk. A small quantity of each homogenized yolk was transferred to a 1.5-ml Eppendorf tube and stored at −20°C until hormone analysis.

Yolk concentrations of testosterone (T), androstenedione (A4) and 5a-dihydrotestosterone (DHT) were determined by radioimmunoassays at the Centre d’Études Biologiques de Chizé following the procedure described in Poisbleau et al. [49], [55]. Due to the fact that A- and B-eggs vary in size and mass in this species [31], a higher hormone concentration in A-eggs than in B-eggs does not necessarily mean a higher hormone quantity for the former. We therefore calculated the yolk hormone amount per yolk (in ng) by multiplying yolk mass (in g) and yolk hormone concentration (in pg/mg).

### Statistical Analysis

All dates, periods of time and adult masses followed a normal distribution (Kolmogorov-Smirnov tests, all *P*>0.303). We obtained arrival dates for only some of the males and females. We therefore analyzed whether time (i.e. arrival date, capture date and the time elapsed between these two dates) influenced other adult parameters separately from the other statistical tests. The within-pair correlations in arrival dates and body masses were tested with a Pearson’s correlation. The effect of female arrival date on the date of laying onset (A-egg laying date) and on the time elapsed between arrival and laying onset were tested using General Linear Models (GLMs) with female arrival date as a covariate. The effect of time on female body mass at laying onset and male body mass few days after laying was also tested using GLMs with each of these three times as covariate.

Egg mass, yolk and albumen masses were normally distributed for A-eggs, for B-eggs, for entire clutches and as ratios between A- and B-eggs (Kolmogorov-Smirnov tests, all *P*≥0.214). The concentrations and amounts of the three androgens were normally distributed (Kolmogorov-Smirnov tests, all *P*≥0.159). These three androgens are not equally potent and differ in receptor affinity [58] and their within-clutch relative difference is potentially important for the organism [59]. We therefore first calculated the yolk androgen concentrations and amounts for the entire clutch (A-egg+B-eggs) and the ratios in concentrations and amounts between A- and B-eggs (A-egg/B-eggs) for each androgen separately. As these androgens were also always positively correlated in terms of concentrations and amounts in A-eggs, in B-eggs, in entire clutches and as ratios between A- and B-eggs (Pearson correlations, all *r*>0.269 and *P*<0.039), we afterward performed eight principal component analyses (PCA) and used their first principal component scores (PC1) to describe yolk androgen concentrations and amounts in A-eggs, in B-eggs, in the entire clutch as well as their ratio between A- and B-eggs (see Table S1). The four PC1 for yolk androgen concentrations explained more than 69.9% of variance and the four PC1 for yolk androgen amounts explained more than 75.5% of variance, with the lowest values for the ratios. We looked at the effect of mate quality on different parameters related to egg provisioning for the entire clutch and for the intra-clutch ratio between A-eggs and B-eggs. GLMs had a two-level fixed factor indicating whether the females were paired with the same mate as during the previous breeding season or with a new mate, as well as male body mass, female body mass and laying date as covariates. In addition, we examined the effects of pair stability on the date of laying onset, male body mass and female body mass using GLMs with a two-level fixed factor indicating whether the pairs were already together during the previous breeding season or were new pairs.

All GLMs were initiated with full-factorial interactions, and subjected to backwards stepwise model simplification with a threshold of *P = *0.05. As a measure of effect sizes we used partial Eta-Square values (*η_p_* ^2^; i.e. the proportion of the effect+error variance that is attributable to the effect, see http://web.uccs.edu/lbecker/SPSS/glm_effectsize.html) for the factors and covariates tested with a GLM. The parameter estimates *B* were given when significant (*P*<0.05) to describe the direction of the relationships. Statistical analyses were conducted in IBM SPSS Statistics 20 for Windows.

## Results

### Dates and Body Masses

On average, females arrived at the colony 3.2±2.4 days (mean ± s.d.; *n* = 30 pairs) after their mate: females arrived before their mates in only 8% of pairs. Male and female arrival dates were not significantly correlated within pairs (*r* = 0.317, *P* = 0.088, *n* = 30 pairs). Whether the females were paired with the same mate as during the previous breeding season or with a new mate was not related to either the female arrival date (*P* = 0.194) or the male arrival date (*P* = 0.774). Females that arrived late at the colony also initiated their clutch late (*F*_1,37_ = 9.092, *P* = 0.005, *η_p_* ^2^ = 0.197, *B* = 0.454) even though they reduced the time elapsed between arrival and laying onset (*F*_1,37_ = 9.419, *P* = 0.004, *η_p_* ^2^ = 0.203, *B* = −0.493). Female body mass at laying onset was not influenced by female arrival date at the colony (*P* = 0.880), capture date (*P* = 0.183,) or the time elapsed between these two dates (*P* = 0.665). Male body mass six to eight days after laying was also not influenced by male arrival date at the colony (*P* = 0.168), capture date (*P* = 0.896) or the time elapsed between these two dates (*P* = 0.052). Female and male body masses were not correlated within pairs (*r* = −0.129, *P* = 0.327, *n* = 60 pairs).

### Mate Quality and Egg Provisioning

**Figure 1:**
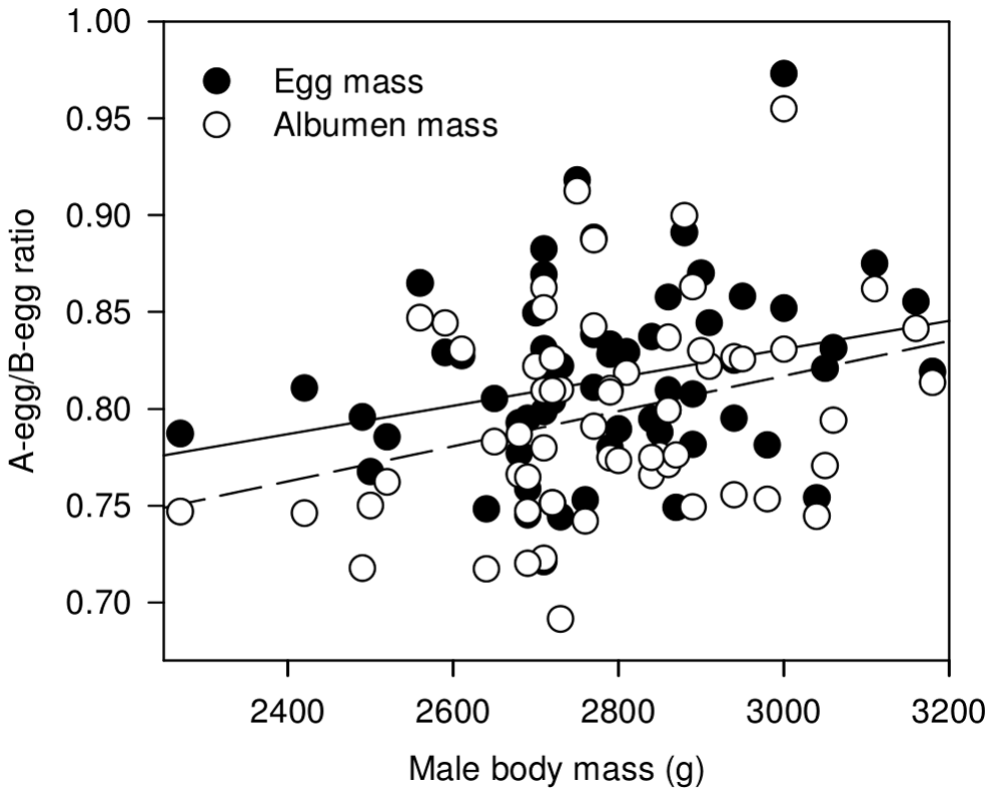
Intra-clutch ratios of egg and albumen masses according to male body mass (in g). The lines are univariate regression models predicting ratios of egg mass (solid line; *P* = 0.027) and albumen mass (dashed line; *P* = 0.016) from male body mass. *n* = 60 clutches.

Egg, yolk and albumen masses of the entire clutch did not vary with male body mass, female body mass, A-egg laying date or whether the females were paired with the same mate as during the previous breeding season or a new mate (Table 1). However, intra-clutch ratios in egg and albumen masses significantly increased (towards equality) with male body mass (Table 1; Figure 1). In other words, the egg mass did not vary for the entire clutches but the relative mass distribution between A- and B-eggs within clutches changed with male body mass. Egg mass allocation increased in A-eggs while it decreased in B-eggs when males were heavier. Moreover, these differences in egg mass were due to differences in albumen mass, but not to differences in yolk mass (see Table 1).

**Table 1:** GLM procedures on egg provisioning parameters.

		Entire clutch			A-egg/B-egg ratio		
Dependent variable	Independent variable	*F*_1,55_	*P*	*η_p_* ^2^	*F*_1,55_	*P*	*η_p_* ^2^
Egg mass	New mate	0.596	0.444	0.011	1.473	0.230	0.026
	Male mass	1.661	0.203	0.029	**5.147**	**0.027**	**0.086**
	Female mass	2.252	0.139	0.039	0.044	0.835	0.001
	Laying date	0.222	0.639	0.004	3.935	0.052	0.067
Yolk mass	New mate	0.578	0.450	0.010	0.088	0.768	0.002
	Male mass	0.110	0.741	0.002	0.305	0.583	0.006
	Female mass	0.102	0.751	0.002	1.319	0.256	0.023
	Laying date	0.088	0.768	0.002	3.420	0.070	0.059
Albumen mass	New mate	1.331	0.254	0.024	1.972	0.166	0.035
	Male mass	1.513	0.224	0.027	**6.176**	**0.016**	**0.101**
	Female mass	2.014	0.161	0.035	0.455	0.503	0.008
	Laying date	0.249	0.619	0.005	2.013	0.162	0.035
Yolk andro. C°	New mate	1.609	0.210	0.028	**5.472**	**0.023**	**0.090**
	Male mass	0.003	0.955	<0.001	1.297	0.260	0.023
	Female mass	0.051	0.821	0.001	0.363	0.549	0.007
	Laying date	**7.405**	**0.009**	**0.119**	0.187	0.668	0.003
Yolk andro. amount	New mate	1.949	0.168	0.034	**4.969**	**0.030**	**0.083**
	Male mass	0.068	0.796	0.001	0.504	0.481	0.009
	Female mass	0.108	0.743	0.002	0.051	0.822	0.001
	Laying date	**4.524**	**0.038**	**0.076**	1.452	0.233	0.026

The results of the GLM procedures on egg provisioning parameters (dependent variables) are given for the entire clutch and for the intra-clutch ratio between A-egg and B-egg. Whether the females were paired with the same mate as during the previous breeding season or with a new mate is a fixed factor and male body mass, female body mass and A-egg laying date are covariates. Values of yolk androgen (andro.) concentrations (C°) and amounts were obtained from principal component analysis (PCA; see methods). *n* = 60 clutches. Significant *P*-values, *P*<0.05, are marked in bold.

**Figure 2:**
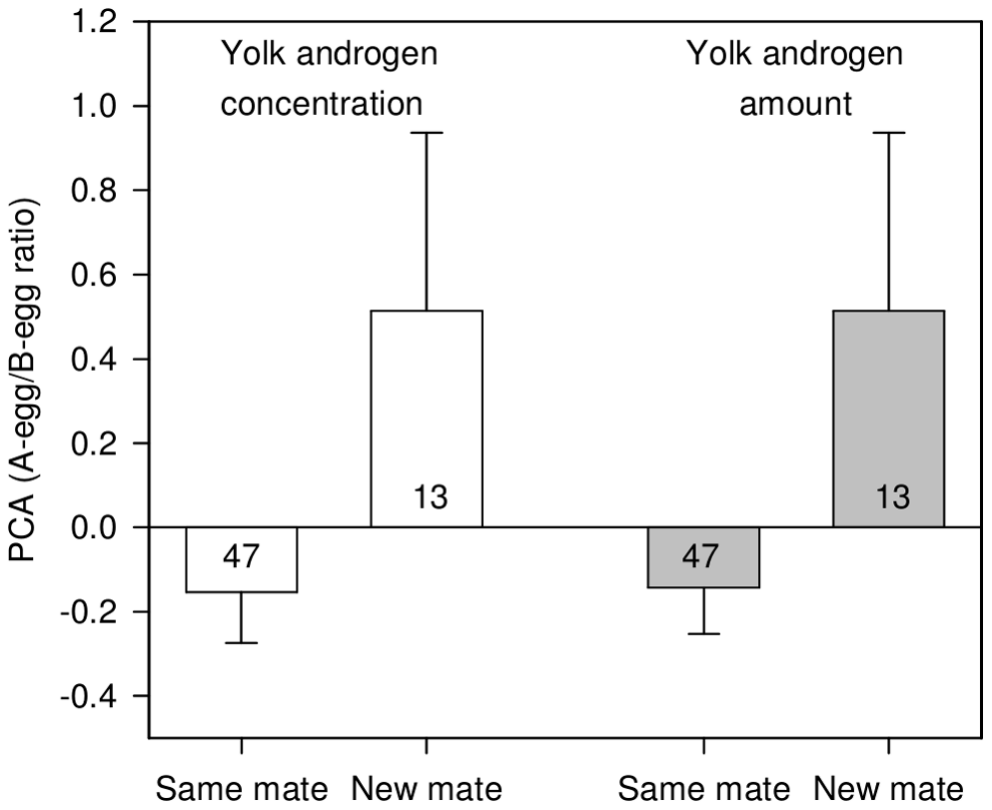
Intra-clutch ratios of yolk androgen concentrations and amounts. The proportion of variance (mean ± s.e.) in intra-clutch ratios of yolk androgen concentrations and yolk androgen amounts between A- and B-eggs is represented according to whether the females were paired with the same mate as during the previous breeding season or with a new mate (*P* = 0.023 for yolk androgen concentrations and *P* = 0.030 for yolk androgen amounts). Values of the ratios in yolk androgen concentrations and amounts were obtained from principal component analysis (PCA; see methods). Numbers inside bars represent sample sizes.

Egg yolk androgens did not vary with male body mass and female body mass but significantly changed with A-egg laying date and whether the females were paired with the same mate as during the previous breeding season or a new mate (Table 1). More specifically, the yolk androgen level in the entire clutches increased as the breeding season progressed (*B* = 0.178 for yolk androgen concentrations and *B* = 0.142 for yolk androgen amounts) but its relative distribution between A- and B-eggs within clutches did not change. In contrast, the yolk androgen levels in the entire clutches did not change with whether the females were paired with the same mate as during the previous breeding season or a new mate but their relative distribution between A- and B-eggs within clutches did change with this parameter. Females paired with a new mate provisioned their clutch with relatively more yolk androgens (in terms of both concentrations and amounts) in the A-egg than the B-egg compared to females paired with the same mate as during the previous breeding season (Figure 2).

In addition, date of laying onset, male body mass and female body mass did not differ between pairs that were already together during the previous breeding seasons and newly formed pairs (*P* = 0.413, *P* = 0.635 and *P* = 0.409).

## Discussion

Females arrived at the breeding site after their mates. Arrival dates were not significantly coordinated within pairs and males and females did not mate assortatively in terms of body mass. These observations suggest that males and females of a pair arrive independently from the wintering places and first meet directly at the breeding place. They demonstrate that male arrival date cannot be used by females as an indicator of mate quality. We therefore assumed that the main ways for females to estimate mate quality were (i) whether or not they were paired with the potential mate during the previous breeding season and (ii) visual assessment of signals such as body mass. We predicted that females should provision their clutch according to the quality of their mate using these two potentially major indicators of mate quality in penguins. We showed that female southern rockhopper penguins provisioned their eggs in masses and androgens differently within a clutch according to the quality of their mate (effect on the ratio between A- and B-eggs). However, surprisingly, the total clutch provisioning in masses and androgens did not vary in relation to mate quality, at least with the two indicators of mate quality we measured (no effect on the total for A- and B-eggs together).

The effects of egg mass and yolk androgens appear to be context-dependent and to be beneficial under sibling competition for birds (reviewed in [60], [61]), including southern rockhopper penguins [17], [31]. In this species, the A-chick commonly dies of starvation within days after hatching when it hatches in a nest with a sibling (i.e. with a B-chick). Indeed, size hierarchy generally favors the oldest and biggest chick(s) [26], [27], which is usually the B-chick in crested penguins. However, when they do not have a sibling, A-chicks show similar growth and survival rates to B-chicks [31]. Moreover, when they do not have a sibling, both A- and B-chicks show a slight decrease in growth and survival when eggs have high levels of yolk androgens [17]. These observations show that egg mass and yolk androgen levels by themselves do not noticeably influence the growth and survival of southern rockhopper penguin chicks, and do not improve the survival of one clutch compared to other clutches. Conversely, within clutches, southern rockhopper penguin chicks from heavy eggs and from eggs with high yolk androgen levels have a faster growth and a higher survival than their siblings from light eggs with low yolk androgen levels (see introduction). In this context, the intra-clutch ratio in mass and androgen levels might be a major driver for the survival of either only one, or of both chicks of the clutch. Our result that total clutch provisioning did not vary with mate quality but that relative intra-clutch allocation in resources did may imply that intra-clutch allocation in resources is more important for female fitness in southern rockhopper penguins than total clutch provisioning. It is therefore probably more adaptive for females in species with brood reduction to modulate their intra-clutch allocation in mass and androgens than to change their total clutch provisioning according breeding conditions such as mate quality.

Females paired with a heavy mate laid less dimorphic clutches than females paired with a low body mass mate. This was more specifically due to changes in albumen mass. In semi-altricial seabirds such as penguins, increases in egg size mainly reflect increases in albumen mass: as egg mass increases, albumen mass increases disproportionally more than expected by isometry (reviewed in [13]). Albumen provides mineral ions but mainly proteins and water for the developing embryo [62], [63]. Since albumen is entirely used up by hatching, it is the protein content in the albumen that limits structural growth of the chick [13]. However, in semi-altricial seabirds, increases in egg size mainly reflect increases in the water content of the albumen [13]. This indicates that the water content of A-eggs proportionally increased with the increasing male body mass compared to the water content of B-eggs (see Table S2). Williams, who also noted a generally positive relationship between egg size and offspring size, growth and survival in the literature on seabirds [13], [64], proposed that a high level of water content could be adaptive [13]. The fact that a reduction in albumen content depresses offspring size, growth and survival [65], [66] also corroborates this adaptive value. Egg size *per se* also may have a significant positive impact on incubation position, temperature and duration ([32] but see [67]). Our observations suggest that females differently allocate water according to their mate quality and that this differential resource allocation might play a role on several components of the brood reduction mechanism observed for this species.

The difference in yolk androgen levels between A- and B-eggs was lower for females paired with a new mate than for females paired with the same mate as during the previous breeding season. This difference results from lower androgen levels in B-eggs from females paired with a new mate than from females paired with the same mate (see Table S2). Since elevated yolk androgen levels in southern rockhopper penguin eggs enhance chick growth and survival in the presence of a sibling [17], we assume that A-chicks have higher probabilities to survive with their B-chick siblings in clutches with a new mate than in clutches with the same mate as during the previous season. This result does not follow our initial prediction on mate quality. This could suggest that new mates are of a higher quality than their previous mate and/or that their chicks are more valuable (differential allocation hypothesis [5], [68]). Nevertheless, the fact that new mates did not have higher body mass than previously known mates does not corroborate this hypothesis. Alternatively, it could mean that females have other reasons than mate quality to increase the chance of fledging two chicks. Females mated with new mates might increase their parental effort to improve the chances of retaining their mate for the subsequent breeding season. For species that exhibit a strong social monogamy and mate fidelity such as southern rockhopper penguins, pair stability may be advantageous by preventing the costs of forming a new pair (pair-bond investment hypothesis [69]). Indeed, finding another mate is costly in energy and time and might lead to decreased reproductive success [70]–[73], even though we did not observe any delay in laying date for females paired with a new male. Moreover, the risk of not finding a new mate also exists [70].

The increase in yolk androgen levels with laying date also deserves some attention. In a previous study on the same population, late-laid B-eggs had higher androgen levels than early-laid B-eggs while no significant difference was observed between early and late clutches for A- eggs [49]. In the present study, we noted an increase in yolk androgen levels for both A-eggs and B-eggs (see Table S2), and subsequently for the total clutches but not for the within-clutch ratio. This difference between the two studies may be explained by the experimental designs. Only two dates (early and late) were sampled in 2007 [49] while the sampling was carried out during the entire laying period in 2010 (present study). Moreover, environmental conditions (temperatures, food availability, nest density…) are likely to have differed between the two breeding seasons and may have led to different patterns of hormone deposition [60], [74]–[78]. Whether females influence the chance to fledge at least one chick (brood reduction *via* the within-clutch ratio in androgen levels) or the chance to fledge the whole clutch (*via* the androgen deposition into both eggs) according to the laying date and environmental conditions still have to be investigated in southern rockhopper penguins.

We showed for the first time that females paired with new mates or heavier mates reduce intra-clutch differences in resource allocation relative to females paired with mates from the previous year or lighter mates. Interestingly, the total clutch provisioning did not vary with mate quality. Taken together, these two separate results suggest that intra-clutch allocation of resources is more important for female fitness in southern rockhopper penguins than total clutch provisioning. This may have direct and strong fitness consequences, significantly increasing a female’s fitness when her mate is of high quality. The pair could therefore have a higher chance of fledging the whole clutch. While female body mass does not seem to play a significant role in this process, the physiological mechanisms that relate male quality to female egg provisioning still have to be investigated to fully understand how females control the size hierarchy among siblings and the potential impacts on a female’s lifetime reproductive success.

## Supporting Information

Table S1 Results of the 8 principal component analyses (PCA). PCA were performed on yolk testosterone (T), yolk androstenedione (A4) and yolk 5a-dihydrotestosterone (DHT) in terms of concentrations (C°) and amounts for the A-eggs, the B-eggs, the entire clutches and the ratios between A- and B-eggs. Since, for each PCA, the first principal component (PC1) explained a very large proportion of variance and was the only one with an eigenvalue superior to 1, we present only the results relative to PC1. n = 60 clutches, i.e. 60 A-eggs and 60 B-eggs.
(DOCX)

Table S2 GLM procedures on egg provisioning parameters. Results of the GLM procedures on egg provisioning parameters (dependent variables) are given for A-eggs and for B-eggs separately. Whether the females were paired with the same mate as during the previous breeding season or with a new mate is a fixed factor and male body mass, female body mass and laying date are covariates. Values of yolk androgen (andro.) concentrations (C°) and amounts were obtained from principal component analysis (PCA; see methods). *n* = 60 A-eggs and 60 B-eggs. Significant P values, *p*<0.05, are marked in bold.
(DOCX)
