# Supplementary material for: Females Paired with New and Heavy Mates Reduce Intra-Clutch Differences in Resource Allocation
Source: PLoS One. 2013 Aug 15;8(8):e72136. doi: 10.1371/journal.pone.0072136 (PMC3744535; doi:10.1371/journal.pone.0072136)
Supplement: Table S1 — Results of the 8 principal component analyses (PCA). PCA were performed on yolk testosterone (T), yolk androstenedione (A4) and yolk 5a-dihydrotestosterone (DHT) in terms of concentrations (C°) and amounts for the A-eggs, the B-eggs, the entire clutches and the ratios between A- and B-eggs. Since, for each PCA, the first principal component (PC1) explained a very large proportion of variance and was the only one with an eigenvalue superior to 1, we present only the results relative to PC1. n = 60 clutches, i.e. 60 A-eggs and 60 B-eggs. (DOCX) [file pone.0072136.s001.docx]

**Table S1.** Results of the 8 principal component analyses (PCA).

|  | Eigenvalue of PC1 | Proportion of variance explained by PC1 (%) | Extraction factors | | |
| --- | --- | --- | --- | --- | --- |
|  |  |  | T | A4 | DHT |
| A-egg C° | 2.176 | 72.531 | 0.952 | 0.722 | 0.866 |
| A-egg amount | 2.377 | 79.231 | 0.961 | 0.801 | 0.902 |
| B-egg C° | 2.166 | 72.187 | 0.938 | 0.673 | 0.913 |
| B-egg amount | 2.390 | 79.676 | 0.948 | 0.788 | 0.932 |
| Entire clutch C° | 2.198 | 73.260 | 0.949 | 0.686 | 0.909 |
| Entire clutch amount | 2.441 | 81.368 | 0.959 | 0.804 | 0.935 |
| A-egg/B-egg C° ratio | 2.096 | 69.866 | 0.829 | 0.706 | 0.857 |
| A-egg/B-egg amount ratio | 2.265 | 75.500 | 0.838 | 0.782 | 0.880 |

PCA were performed on yolk testosterone (T), yolk androstenedione (A4) and yolk 5a-dihydrotestosterone (DHT) in terms of concentrations (C°) and amounts for the A-eggs, the B-eggs, the entire clutches and the ratios between A- and B-eggs. Since, for each PCA, the first principal component (PC1) explained a very large proportion of variance and was the only one with an eigenvalue superior to 1, we present only the results relative to PC1. *n* = 60 clutches, i.e. 60 A-eggs and 60 B-eggs.
