# Supplementary material for: Females Paired with New and Heavy Mates Reduce Intra-Clutch Differences in Resource Allocation
Source: PLoS One. 2013 Aug 15;8(8):e72136. doi: 10.1371/journal.pone.0072136 (PMC3744535; doi:10.1371/journal.pone.0072136)
Supplement: Table S2 — GLM procedures on egg provisioning parameters. Results of the GLM procedures on egg provisioning parameters (dependent variables) are given for A-eggs and for B-eggs separately. Whether the females were paired with the same mate as during the previous breeding season or with a new mate is a fixed factor and male body mass, female body mass and laying date are covariates. Values of yolk androgen (andro.) concentrations (C°) and amounts were obtained from principal component analysis (PCA; see methods). n = 60 A-eggs and 60 B-eggs. Significant P values, p<0.05, are marked in bold. (DOCX) [file pone.0072136.s002.docx]

**Table S2.** GLM procedures on egg provisioning parameters.

|  |  | A-egg | | | B-egg | | |
| --- | --- | --- | --- | --- | --- | --- | --- |
| Dependent  variable | Independent variable | *F*_1,55_ | *p* | *η_p_*² | *F*_1,55_ | *p* | *η_p_*² |
| Egg mass | New mate | 0.883 | 0.351 | 0.016 | 0.271 | 0.605 | 0.005 |
|  | Male mass | 3.201 | 0.079 | 0.055 | 0.399 | 0.530 | 0.007 |
|  | Female mass | 1.391 | 0.243 | 0.025 | 2.944 | 0.092 | 0.051 |
|  | Laying date | 1.072 | 0.305 | 0.019 | 0.033 | 0.857 | 0.001 |
| Yolk mass | New mate | 0.454 | 0.503 | 0.008 | 0.503 | 0.481 | 0.009 |
|  | Male mass | 0.222 | 0.639 | 0.004 | 0.014 | 0.906 | < 0.001 |
|  | Female mass | 0.564 | 0.456 | 0.010 | 0.048 | 0.827 | 0.001 |
|  | Laying date | 1.001 | 0.321 | 0.018 | 0.097 | 0.756 | 0.002 |
| Albumen mass | New mate | 1.712 | 0.196 | 0.030 | 0.787 | 0.379 | 0.014 |
|  | Male mass | 3.192 | 0.080 | 0.055 | 0.265 | 0.609 | 0.005 |
|  | Female mass | 0.914 | 0.343 | 0.016 | 3.275 | 0.076 | 0.056 |
|  | Laying date | 0.780 | 0.381 | 0.014 | 0.001 | 0.972 | < 0.001 |
| Yolk andro. C° | New mate | 0.344 | 0.560 | 0.006 | **5.143** | **0.027** | **0.086** |
|  | Male mass | 0.258 | 0.614 | 0.005 | 0.250 | 0.619 | 0.005 |
|  | Female mass | 0.087 | 0.769 | 0.002 | 0.271 | 0.605 | 0.005 |
|  | Laying date | **4.274** | **0.043** | **0.072** | **6.603** | **0.013** | **0.107** |
| Yolk andro. amount | New mate | < 0.001 | 0.992 | < 0.001 | **4.017** | **0.050** | **0.068** |
|  | Male mass | 0.018 | 0.894 | < 0.001 | 0.215 | 0.645 | 0.004 |
|  | Female mass | 0.061 | 0.804 | 0.006 | 0.102 | 0.750 | 0.002 |
|  | Laying date | **4.672** | **0.035** | **0.078** | 3.177 | 0.080 | 0.055 |

Results of the GLM procedures on egg provisioning parameters (dependent variables) are given for A-eggs and for B-eggs separately. Whether the females were paired with the same mate as during the previous breeding season or with a new mate is a fixed factor and male body mass, female body mass and laying date are covariates. Values of yolk androgen (andro.) concentrations (C°) and amounts were obtained from principal component analysis (PCA; see methods). *n* = 60 A-eggs and 60 B-eggs. Significant P values, *p* < 0.05, are marked in bold.
